# Interleukin-35 -producing B cells rescues inflammatory bowel disease in a mouse model via STAT3 phosphorylation and intestinal microbiota modification

**DOI:** 10.1038/s41420-023-01366-5

**Published:** 2023-02-17

**Authors:** Minxiang Xie, Yuzhen Zhu, Yunjiao Zhou, Qiao Wang, Erli Gu, Yiwei Chu, Luman Wang

**Affiliations:** 1 Department of Immunology, School of Basic Medical Sciences, and Institutes of Biomedical Sciences, Fudan University, Shanghai, 200032 China; 2 Key Laboratory of Medical Molecular Virology (MOE/NHC/CAMS), School of Basic Medical Sciences, Fudan University, Shanghai, 200032 China; 3 Department of gastroenterology, Jing’an District Central Hospitals, Fudan University, Shanghai, China; 4 Biotherapy Research Center, Fudan University, Shanghai, 200032 China; 5 Department of Endocrinology and Metabolism, Shanghai Fifth People’s Hospital, Fudan University, Shanghai, China

**Keywords:** Cell death and immune response, Inflammatory bowel disease

## Abstract

Interleukin-35 (IL-35)-producing B cells (IL-35^+^B cells) play an important role in diseases, and the expansion of IL-35^+^ immune cells have been observed in inflammatory bowel disease (IBD). However, how IL-35^+^B cells function and the manner in which they perform their roles remain unclear. In this study, human samples and animal models were used to confirm the expansion of IL-35^+^B cells during IBD. In addition, by using *il12a*^*−/−*^ and *ebi3*^*−/−*^ mice, we demonstrated that the regulatory role of B cells in IBD depends on IL-35. Mechanically, IL-35^+^B cells can promote its own expansion through endocrine actions and depend on the transcription factor signal transducer and activator of transcription 3. Interestingly, we found that the diversity of intestinal microbes and expression of microbial metabolites decreased during IBD. IL-35^+^B cells promote the high expression of indoleacetic acid (IAA), and exogenous metabolite supplementation with IAA can further promote the expansion of IL-35^+^B cells and rescues the disease. This study provides a new concept for the regulatory model of B cells and a new approach for the treatment of IBD.

## Introduction

Interleukin-35 (IL-35) is a heterodimeric cytokine that consists of two monomers, IL12a and Ebi3. It is a recently identified cytokine of the family, which plays a key role in the suppressive function of immune cells [1, 2]. The IL-35-producing B cells (IL-35^+^B cells) is the most recently described B cell subset and can exert inhibitory effects through both monomers and as a whole [3–5]. The IL-35^+^B cells and IL-35 play an important role in immune-related diseases. For instance, mice deficient in B cell derived IL-35 develop exacerbated experimental autoimmune uveoretinitis and experimental allergic encephalomyelitis (EAE) [4, 5]. In hepatitis B virus infection, it has been suggested that IL-35 modulates the balance between regulatory and effector lymphocytes with disease outcome depending on the stage of the disease [6]. In inflammatory bowel disease (IBD), studies have shown that IL-35 is abnormally expressed in patients [7, 8], but the role of IL-35^+^B cells in IBD has not been reported. IL-35 functions through receptors, but it has various receptors. In T or B cells, IL-35 is thought to signal through the IL12Rβ2/lL12Rβ2, IL12Rβ2/gp130, gp130/gp130, and IL-12Rβ2/IL27Rα receptors [5, 9]. Upon binding to cognate receptors, the receptor-associated Janus kinases (Jak1, Jak2, and Tyk2) are activated, providing phosphotyrosine-docking sites that recruit specific members of the signal transducer and activator of transcription (STAT) transcription factors [10, 11]. However, there are many unknown aspects about IL-35^+^B cells, including how IL-35^+^B cells interact with the disease microenvironment and orchestrates the immune response to maintain homeostasis.

Microbes and their metabolites play a critical role in IBD, *Ruminococcaceae*, *Enterobacteriaceae*, and *Escherichia coli* are highly expressed in the intestinal tract of patients with IBD, while the expression of *Lachnospiraceae*, *Coriobacteriaceae*, and *Bifidobacteriaceae* are decreased in the intestinal tract of these patients [12, 13]. Toll-like receptor ligand and microbe metabolites, short chain fatty acid (SCFA), conjugated linoleic acid, and indoleacetic acid (IAA) can reshape and reprogram immune cells, including B cells [14, 15]. For example, TLR5 is a key receptor for the maintenance of B-1a B cells. And flagellin, the TLR5 ligand on the surface of intestinal microbes, is an indispensable factor for the remodeling of this B cell subtype [16]. The SCFA butyric acid increased the anti-tumor effect in CD8^+^T cells by increasing the expression and function of the inhibitor of DNA binding 2 (ID2) [15]. IAA as a microbe metabolite can induce Th22 cells to produce more IL-22 by regulating the islet regeneration protein, thus performing a negative regulatory role [17]. Importantly, recent reports have indicated that IAA and lipopolysaccharide (LPS) can induce IL-35^+^B cell expression in vitro and in vivo [18]. However, the relationship between microbe metabolites and IL-35^+^B cells in IBD remains unknown.

Our results suggest that IL-35^+^B cell expansion during IBD relies on EBI3 and IL12A to perform their inhibitory functions to maintain intestinal homeostasis for disease remission. During the disease, both the microbe diversity and microbe metabolite expression were decreased. After the metabolites were supplemented with IAA the expression of IL-35^+^B cells was significantly increased, and the disease was effectively treated.

## Results

### IL-35^+^B cell expansion in the intestinal tract of patients with IBD and dextran sulfate sodium induced colitis in mice

**Fig. 1:**
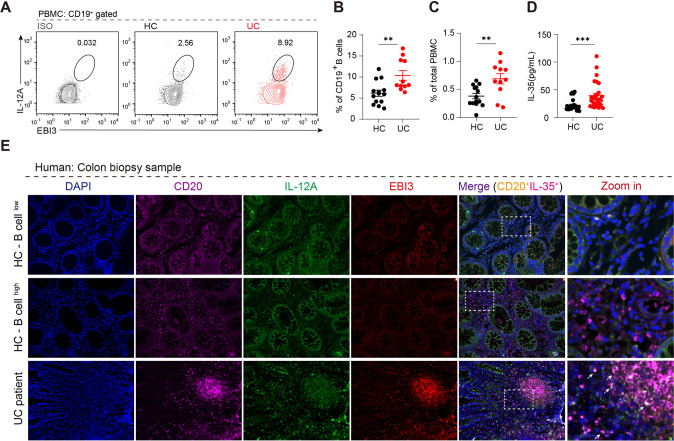
IL-35 producing B cells are preferentially upregulated in patients with ulcerative colitis (UC). **A** Expression levels of IL-35 in B cells from the peripheral blood from patients with UC and healthy controls were detected by flow cytometry, representative IL-12A^+^ EBI3^+^ expression in CD19^+^ B cells isolated from the healthy control group (*n* = 14); UC patient group (*n* = 11). **B** Dots represent IL-12A^+^ EBI3^+^ cell frequencies in isolated CD19^+^ B cells and total peripheral blood mononuclear cells (PBMCs). **C** Dots represent IL-12A^+^ EBI3^+^ cell frequencies in total PBMCs. **D** IL-35 production in the serum from patients with UC and healthy controls were detected by ELISA (healthy control group *n* = 14; patients with UC group *n* = 14, two representative independent experiments are presented out of three similar experiments performed). **E** Expression levels of IL-35 in CD20^+^ B cells from formalin-fixed paraffin-embedded colon tissue sections from six patients with UC and six healthy controls were determined by immunofluorescence staining. **B**–**D** Two-tailed unpaired Student’s *t*-test. Data are shown as mean ±standard error of the mean (SEM) (*P < 0.05, **P < 0.01, ***P < 0.001, ****P < 0.0001).

To identify the immune system-related profiles of patients with ulcerative colitis (UC), we found a suitable dataset from the Gene Expression Omnibus (GEO) database. According to GSE11223, in samples from patients with active UC, regulatory cytokines such as IL-10 and EBI3 were significantly increased (Fig. S1A). In addition, the gene expression profile was obtained from GSE38713, and the immune cell proportions in the colon of healthy controls and patients with UC were analyzed using xCell. As shown in Fig. S1B, C, compared with the healthy controls B cells were more active in patients with UC. Cytokine analysis of B cells confirmed that Ebi3 expression was positively correlated with B cell activity (Fig. S1D, E). We collected peripheral blood and biopsy samples from 15 patients with UC and flow cytometry and ELISA results showed increased IL-35^+^B cells (IL12a and EBI3) (Fig. 1A–C) and IL-35 expression (Fig. 1D) in the peripheral blood, respectively. Immunofluorescence staining confirmed the expression of IL-35^+^B cells infiltration in the intestine samples of patients with UC (Fig. 1E).

**Fig. 2:**
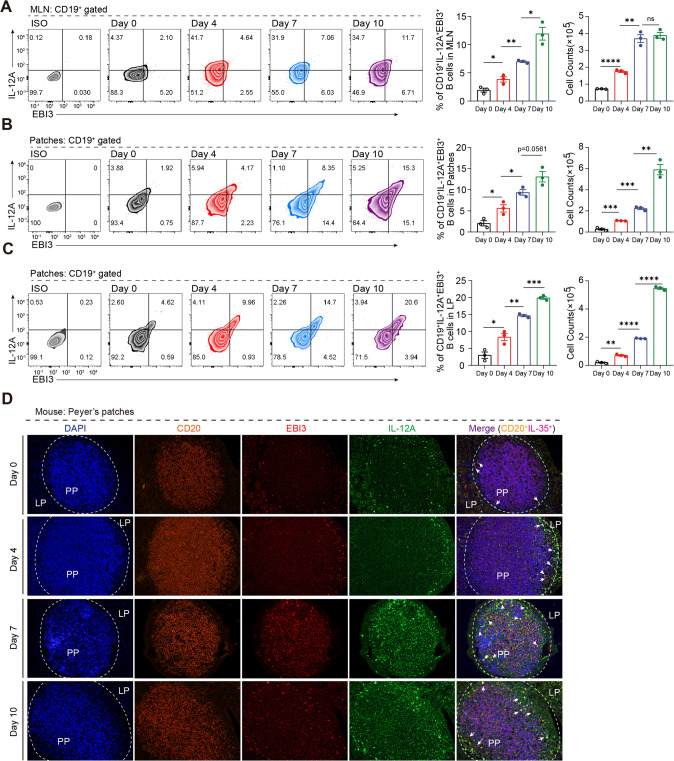
IL-35 producing B cells are the dominant regulatory population during colitis. **A**–**C** Cells were isolated from mesenteric lymph nodes (MLNs) (**A**), Peyer’s patches (Patches) (**B**), and colorectal lamina propria (LP) (**C**) from 2.5% dextran sulfate sodium (DSS)-treated wildtype (WT) mice at day 0, 4, 7, and 10 post-disease onset. The percentage and absolute number of IL-35 producing B cells (CD19^+^ IL-12A^+^ EBI3^+^ cells) were determined by flow cytometry (*n* = 3 per group). **D** Expression levels of IL-35 producing B cells (CD20^+^ IL-12A^+^ EBI3^+^ cells) in formalin-fixed paraffin-embedded Peyer’s patches tissue sections from DSS-treated mice were determined by immunofluorescence staining (*n* = 6 per group). **A**–**D** Two-tailed unpaired Student’s *t*-test. Data are shown as mean ± standard error of the mean (SEM) and are representative of at least three independent experiments (*P < 0.05, **P < 0.01, ***P < 0.001, ****P < 0.0001).

Next, we used a widely accepted 2.5% dextran sulfate sodium (DSS)-induced colitis murine model and performed more detailed intestinal and in vivo experiments to confirm the above results (Fig. S2A). As shown in Fig. S2, the DSS-induced mice exhibited significantly more weight loss (Fig. S2B) and severe disease symptoms as measured by the disease activity index (DAI) score (Fig. S2C). The DSS-treated mice showed shortening of the colon in the acute stage (day 4 and 7) and recovered in the remission stage (day 10) (Fig. S2D). Moreover, the overall survival was decreased after DSS induction (Fig. S2E). To further evaluate the disease severity the degree of intestinal injury was histopathologically assessed and acute colitis in the DSS-induced mice was characterized by epithelial damage, focal crypt injury, goblet cell depletion, and inflammatory cell invasion, which were milder in remission stage (Fig. S2F). We then extracted RNA from the B cells of the Payers patches and the intestinal proteins to detect the cytokine profile. We found that pro-inflammation cytokines such as IL-6, IFN-γ, and TNF-α were increased during acute stage and decreased in remission stage, while anti-inflammation cytokines such as IL-35 (Il12a and Ebi3) were consistently increased throughout, suggesting that IL-35 may play an important role in maintaining homeostasis (Fig. S2G, H). Moreover, we found that the source of IL-35 was mainly the B cells in the intestine (Fig. S2I, J). Importantly, using fluorescence-activated cell sorting and immunofluorescence, we confirmed that IL-35^+^B cells in intestine associated lymph tissues were expanded during inflammation (Fig. 2A–D), which was consistent with the database results from patients with UC. Taken together, these results suggest that B cell derived IL-35 expansion during colitis may play an important role in maintaining intestinal homeostasis.

### B cell rescue of colitis is dependent on IL-35

**Fig. 3:**
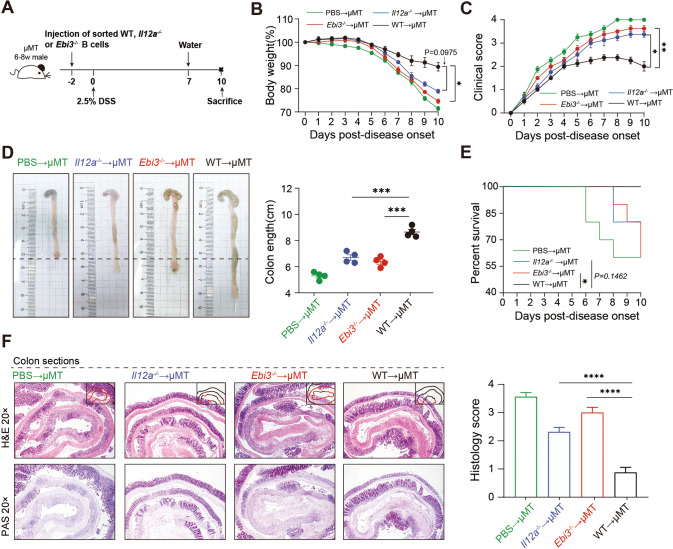
Functional impairment of IL-35 production in B cells leads to severe colitis in a DSS-induced colitis mouse model. **A** CD19^+^ B cells from the spleens of wildtype (WT), *Il12a*^*−/−*^ and/or *Ebi3*^*−/−*^ mice were adoptively transferred into B cell depleted (μMT) mice intravenously 48 h before 2.5% dextran sulfate sodium (DSS) administration. **B** Body weight was calculated (*n* = 5 per group). **C** Disease activity index (DAI) was calculated (*n* = 5 per group). **D** Representative colon sections are shown for each group (left panel). Length of the colon is shown on the bar graph (right panel) (*n* = 5 per group). **E** Survival analysis of each group was calculated (*n* = 5 per group). **F** Representative colon sections stained with hematoxylin and eosin (upper panels) and periodic acid-Schiff (lower panels) are shown for each group. Images are shown at ×20 magnification. Histological sections were blindly scored on a scale of 0–4 to generate a histological score and individual mouse scores are shown with each data point representing a single mouse (*n* = 10 per group, right panel). **B**, **C** Two-way analysis of variance (ANOVA) with Dunnett’s multiple comparisons test. **D**, **F** Two-tailed unpaired Student’s *t*-test. **E** Log-rank (Mantel–Cox) test. Data are shown as mean ± standard error of the mean (SEM) and are representative of at least three independent experiments (*P < 0.05, **P < 0.01, ***P < 0.001, ****P < 0.0001).

We next sought to determine if IL-35 was essential for B cells to maintain intestine homeostasis. B cells were purified from wildtype (WT), *Il12a*^*−/−*^, and *Ebi3*^*−/−*^ mice and transferred into B cell-deficient (μMT) mice (Fig. 3A, S4). Body weight loss was observed in WT transferred group on day 6 and the decrease stopped on day 8. In contrast, *Il12a*^*−/−*^ and/or *Ebi3*^*−/−*^ transferred group began to show significant body weight loss on day 5 and continued to lose weight until the last day of the experiment (Fig. 3B). Furthermore, the *Il12a*^*−/−*^ and/or *Ebi3*^*−/−*^ transferred group showed an increased DAI score, which were significantly higher than WT transferred group (Fig. 3C). The gross pathology of the intestines from each group is shown in Fig. 3D, and the *Il12a*^*−/−*^ and/or *Ebi3*^*−/−*^ transferred group showed apparent shortening and reddening of the colon, which are typical colitis characteristics. The WT transferred group showed milder intestine changes (Fig. 3D). The *Il12a*^*−/−*^ and *Ebi3*^*−/−*^ transferred group had reduced overall survival (Fig. 3E). Histologically, the control mice from the *Il12a*^*−/−*^ and *Ebi3*^*−/−*^ transferred group exhibited the most severe inflammation throughout the entire intestine, with massive loss of crypts and leukocyte infiltration observed on day 7 (Fig. 3F). These results suggest that B cell rescued colitis is dependent on IL-35. Notably, when we induced colitis directly into *Il12a*^*−/−*^ and *Ebi3*^*−/−*^ mice (Fig. S3A), the knockout mice were not found to be more severely affected than the WT mice (Fig. S3B−G). This indicates that only B cell derived IL-35 and EBI3 displayed inhibitory effects in colitis.

### rmIL-35 induces IL-35^+^B cells expansion and rescued colitis

**Fig. 4:**
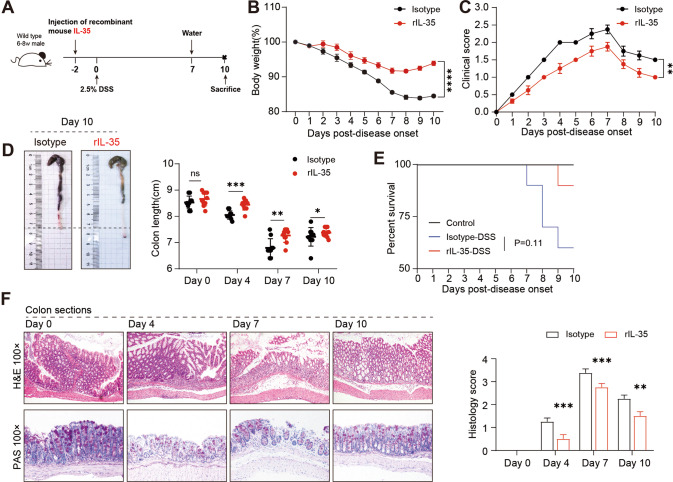
Recombinant IL-35 supplement suppresses severe colitis symptoms in a DSS-induced colitis mouse model. **A** Wildtype (WT) mice were pre-treated with recombinant IL-35 or IgG isotype intravenously 48 h before 2.5% dextran sulfate sodium (DSS) administration. **B** Body weight was calculated (*n* = 4 per group). **C** Disease activity index (DAI) was calculated (*n* = 4 per group). **D** Representative colon sections are shown for each group (left panel). Length of the colon is shown on the bar graph (right panel) (*n* = 4 per group). **E** Survival analysis of each group was calculated (*n* = 10 per group). **F** Representative colon sections stained with hematoxylin and eosin (upper panels) and periodic acid-Schiff (lower panels) are shown for each group. Images are shown at ×100 magnification. Histological sections were blindly scored on a scale of 0–4 to generate a histological score and individual mouse scores are shown with each data point representing a single mouse (*n* = 4 per group). **B**, **C** Two-way analysis of variance (ANOVA) with Dunnett’s multiple comparisons test. **D**, **F** Two-tailed unpaired Student’s *t*-test. **E** Log-rank (Mantel–Cox) test. Data are shown as mean ± standard error of the mean (SEM) and are representative of at least three independent experiments (*P < 0.05, **P < 0.01, ***P < 0.001, ****P < 0.0001).

**Fig. 5:**
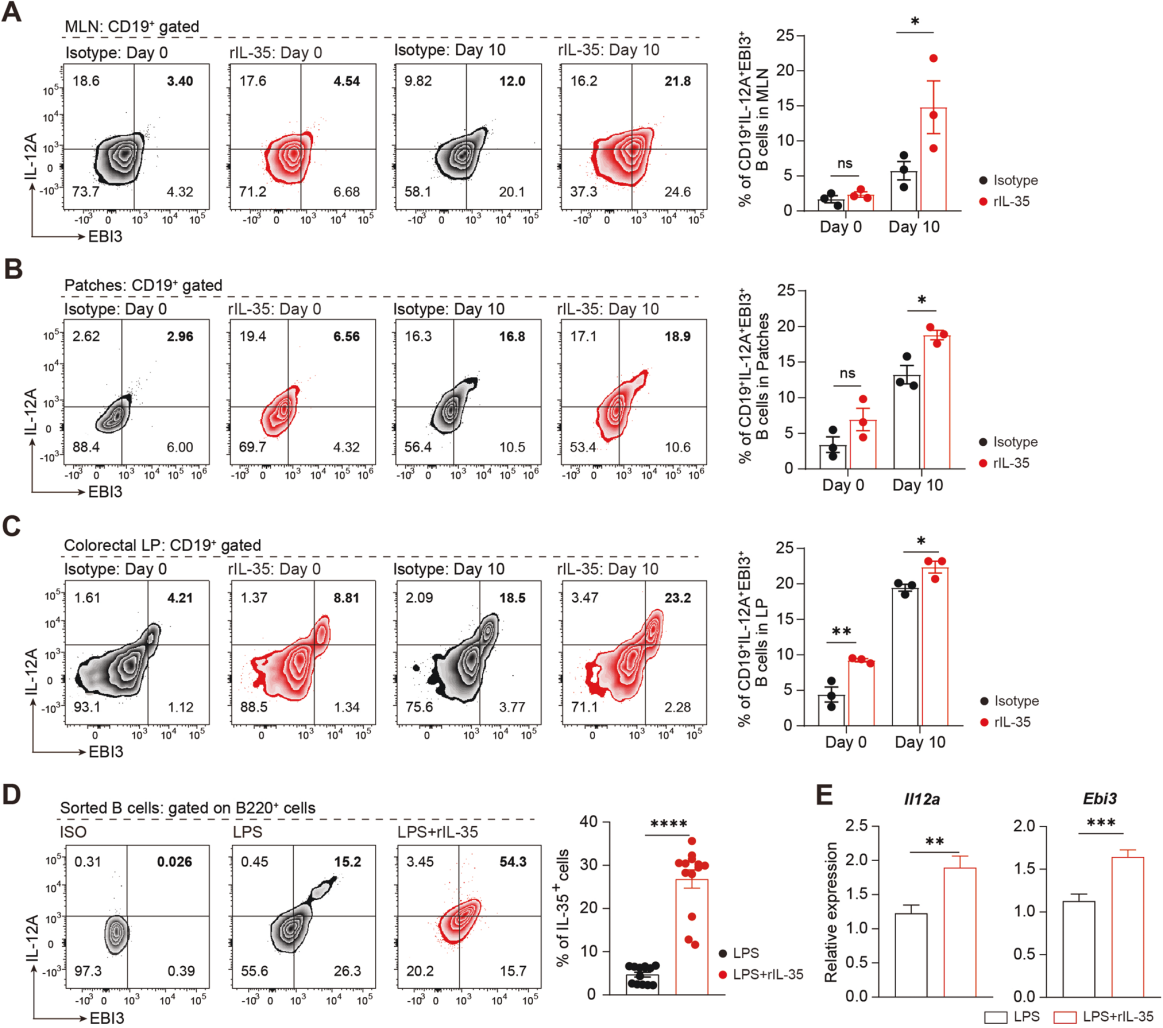
Supplemented IL-35 attenuates severe colitis by skewing the B cell compartment in favor of IL-35 production. **A**–**C** Cells were isolated from mesenteric lymph nodes (MLNs) (**A**), Peyer’s patches (Patches) (**B**) and lamina propria (LP) (**C**) of mice pre-treated with recombinant IL-35 or IgG at day 0 and 10 post-disease onset. The percentage of IL-35 producing B cells (CD19^+^IL-12A^+^EBI3^+^ cells) was determined by flow cytometry (*n* = 3 per group). **D** Percentage of IL-35 producing B cells (B220^+^ IL-12A^+^ EBI3^+^ cells) with or without 20 ng/mL rIL-35 stimulation was detected by flow cytometry (*n* = 13 per group). **E** Production of IL-35 (*Il12a* and *Ebi3*) in B cells with or without 20 ng/mL rIL-35 stimulation was detected by RT-PCR (*Il12a*: lipopolysaccharide (LPS) group *n* = 9; LPS + rIL-35 group *n* = 15; *Ebi3*: LPS group *n* = 9; LPS + rIL-35 group *n* = 14). **A**–**E** Two-tailed unpaired Student’s *t*-test. Data are shown as mean ± standard error of the mean (SEM) and are representative of at least three independent experiments (*P < 0.05, **P < 0.01, ***P < 0.001, ****P < 0.0001).

We further investigated whether IL-35 or IL-35^+^B cells could be used to treat colitis. The mice were injected intraperitoneally with recombined murine IL-35 (rmIL-35, 400 ng/mouse) 48 h before DSS induction of colitis (Fig. 4A). Mice that received rmIL-35 exhibited milder symptoms in the intestines than mice that received isotype IgG1. The rmIL-35 treated mice experienced almost no body weight loss and diarrhea (Fig. 4B, C). The gross pathology of the intestines, histological staining, and overall survival showed that injection with rmIL-35 significantly alleviated colitis (Fig. 4D–F). Intriguingly, rmIL-35 promoted IL-35^+^B cell expansion in the gut-associated lymphoid tissue (GALT) (Fig. 5A–C). In addition, the in vitro experiments showed that the proportion of IL-35^+^B cells increased after the addition of rmIL-35 in the cultured system (Fig. 5D, E). This data strongly suggests that IL-35 promotes the IL-35^+^B cells expansion and consequently alleviated the DSS-induced colitis, thus exhibiting a therapeutic effect.

### Autocrine IL-35 promoting IL-35^+^B cells through STAT3 phosphorylation

**Fig. 6:**
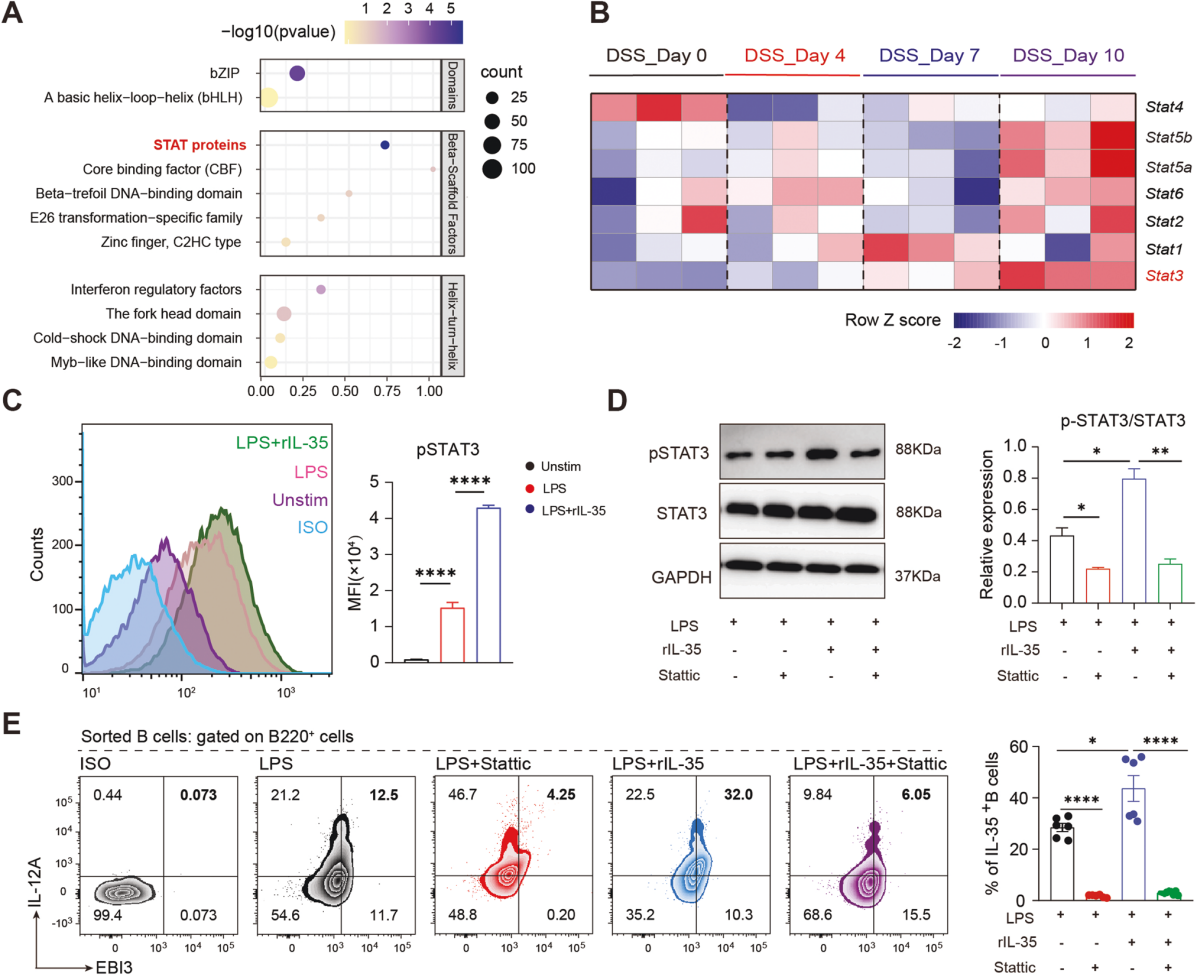
Autocrine action of IL-35 producing B cells depends on the STAT3 pathway. **A** Bubble diagram shows transcription factor enrichment analysis ranked by significance in CD19^+^ B cells isolated from Peyer’s patches of colitis induced mice at day 0, 4, 7, and 10 post-disease onset (*n* = 6 per group). **B** Heatmap shows the expression of the STAT family proteins in CD19^+^ B cells isolated from Peyer’s patches of colitis induced mice at day 0, 4, 7, and 10 post-disease onset. Data are normalized and shown in the format of transcripts per million. Genes that are highly correlated with the expression levels of IL-35 in colitis are highlighted (*n* = 6 per group). **C** The expression of p-STAT3 in CD19^+^ B cells after 72 h stimulation with or without rIL-35 are shown as mean fluorescence intensity (MFI) (*n* = 3 per group). Bar graphs represent the MFI of the groups (right panel). **D** Western blot of B cells stimulated under IL-35 producing B cells culture conditions with or without 10 nM STAT3 inhibitor (stattic, 10 ng/mL) (*n* = 3 per group). **E** Percentage of IL-35 producing B cells (B220^+^ IL-12A^+^ EBI3^+^ cells) under IL-35 producing B cells culture conditions with or without 10 nM STAT3 inhibitor (stattic) was detected by flow cytometry (lipopolysaccharide (LPS) group *n* = 6; LPS + rIL-35 group *n* = 6; LPS + stattic group *n* = 6; LPS + rIL-35+stattic group *n* = 9). **C**–**E** Two-tailed unpaired Student’s *t*-test. Data are shown as mean ± standard error of the mean (SEM) and are representative of at least three independent experiments (*P < 0.05, **P < 0.01, ***P < 0.001, ****P < 0.0001).

As shown in Fig. 5, IL-35 has positive feedback to promote IL-35^+^B cells in vivo and in vitro, thus maintaining tissue homeostasis. Therefore, further exploration into the molecular mechanism of autocrine action of IL-35^+^B cells was warranted. RNA-seq revealed an overall increase in the expression of the STAT family of B cells during colitis, and the STAT3 expression was most consistent with that of IL-35 (Fig. 6A, B). We analyzed the phosphorylation of STAT3 by flow cytometry and western blot and found that the phosphorylation of B cells was significantly increased after stimulation with IL-35 (Fig. 6C, D). Stattic is a STAT3 inhibitor, which can effectively inhibit the phosphorylation of STAT3 (Fig. 6D). LPS can induce the expression of IL-35 in B cells, but the expression of IL-35^+^B cells was significantly reduced after the addition of Stattic, and the addition of exogenous IL-35 was not able to reverse this reduction. In addition, rmIL-35 can also promote the increase of IL-10^+^B cells, but was inhibited by Stattic (Fig. S5A, B), indicating that the induction of IL-35 in B cells is biased towards the regulatory phenotype. Taken together, these results suggest that after inhibition of STAT3 phosphorylation, autocrine IL-35 cannot promote the expression of IL-35^+^B cells, and IL-35^+^B cells can self-promote through STAT3 phosphorylation.

### IL-35^+^B cells modify the intestinal microbiota and their metabolites

**Fig. 7:**
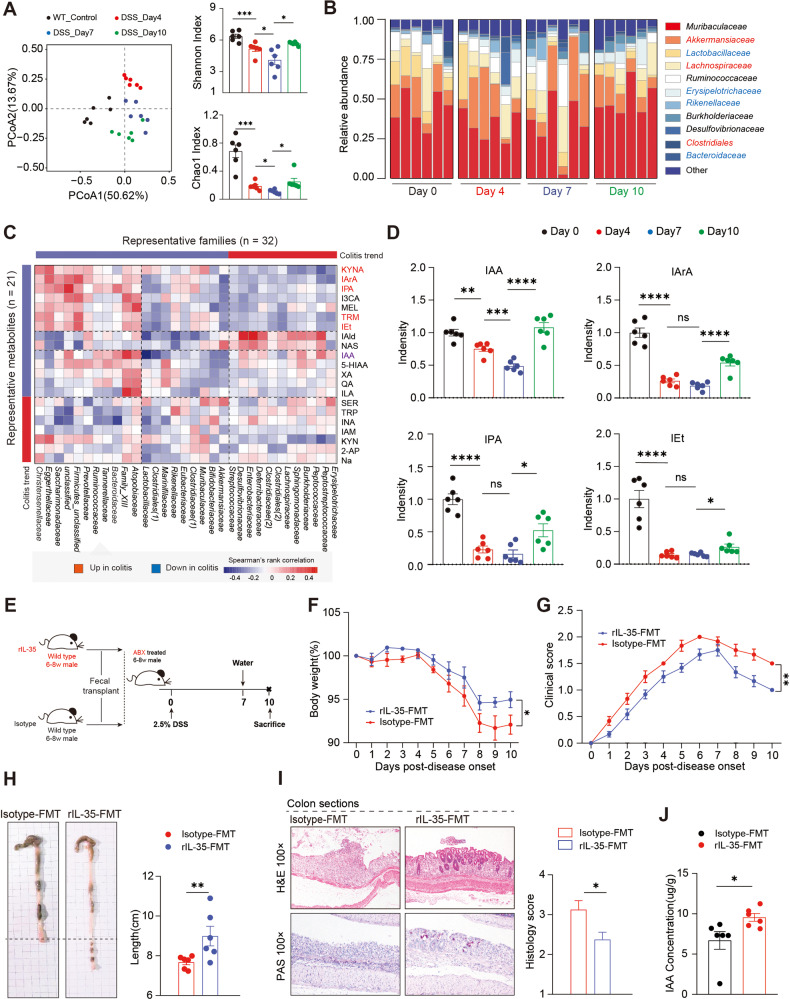
Gut microbiota and tryptophan metabolite profiles in colitis. **A** Fecal samples (*n* = 24) were collected from 2.5% dextran sulfate sodium (DSS)-treated wildtype (WT) mice at day 0, 4, 7, and 10 post-disease onset and isolated genomic DNA was sequenced for the 16 S rRNA gene at the V3-V4 region. Principal coordinate analysis, Shannon index, and Chao1 index were calculated (*n* = 6 per group). **B** The taxonomic variations of microbial communities across mice of different post-disease onset were analyzed and shown as a stack bar plot (*n* = 6 per group). **C** Tryptophan metabolites in the 24 fecal samples were detected by high performance liquid chromatography (*n* = 6 per group). The correlations between the 21 metabolites and 32 significant microbiota families (shown in **B**) were calculated using spearman’s rank correlation analysis. Microbiota families or metabolites which were upregulated in colitis were clustered into the red color module, and downregulated ones were clustered into the blue color module. **D** Metabolites concentration of differential metabolites (IAA, IArA, IPA, and IEt) which were highlighted in **C** are shown as density in the bar plots (*n* = 6 per group). **E** Experimental scheme outlining the water schedule, duration of diet, and microbial transplantation. **F** Body weight was calculated (*n* = 6 per group). **G** Disease activity index (DAI) was calculated (*n* = 6 per group). **H** Representative colon sections are shown for each group. Length of the colon is shown on the bar graph (*n* = 6 per group). **I** Representative colon sections stained with hematoxylin and eosin (upper panel) and periodic acid-Schiff (lower panel) are shown for each group. Images are shown at ×100 magnification (*n* = 8 in each group). **J** The concentration of IAA after FMT. **A**, **D**, **H**, **I**, **J** Two-tailed unpaired Student’s *t*-test. **C** Spearman’s rank correlation coefficient. **F**, **G** Two-way analysis of variance (ANOVA) with Dunnett’s multiple comparisons test. Data are shown as mean ±standard error of the mean (SEM) and are representative of at least three independent experiments (*P < 0.05, **P < 0.01, ***P < 0.001, ****P < 0.0001).

Previous studies have demonstrated that the cytokines that alleviate colitis are closely dependent on the intestinal microbiota [19–22]. The mutual crosstalk between the cytokines and intestinal microbiota may lead to the hypothesis that IL-35^+^ B cell are closely related to the modification of intestinal microbiota. Therefore, we characterized the microbial composition of fecal samples collected from mice with DSS-induced colitis on day 0, 4, 7, and 10 using 16 S ribosomal RNA (rRNA) gene sequencing analyses. Figure 7A, B show that the diversity of the intestinal microbiota gradually decreased and then increased during the remission stage of the disease. Generally, the intestinal microbiota immunomodulatory function depends on their metabolites [23, 24]. Therefore, to further explain the association between IL-35^+^B cell and the intestinal microbiota, we performed tryptophan (indole) targeted metabolomic tests. We measured 21 differential metabolites and analyzed their correlation with 32 intestinal microbiotas as shown in Fig. 7C. The diversity of microbial metabolites was consistent with that of the microorganisms (Fig. 7C, S6), and it is worth noting that, the expression of IAA, Indole-3-propionic acid (IPA), Indole-3-acrylic acid (IArA), and Tryptophol (Iet) were decreased during the acute stage and recovered during the remission stage. Among them, the expression of IAA consistently followed the changes of disease course (Fig. 7D). We hypothesized that IL-35^+^B cells could modify the diversity of gut microbiota and metabolites, thereby alleviating disease. A fecal microbiota transplantation (FMT) was performed to confirm our hypothesis. Recipient mice were divided into two groups. The first group was stimulated with rmIL-35 and then their stool was transplanted into ABX-treated mice (rmIL-35); and the second group was stimulated with the isotype control and then their stool was transplanted into ABX-treated mice (isotype) (Fig. 7E). We measured the body weight and DAI every day, and the colon length was measured with the histological staining. The rmIL-35 FMT group shown significantly reduced colitis symptoms (Fig. 7F–I) and we found that IAA was increased after IL-35 treatment (Fig. 7J). These results suggest that the presence of IL-35 could regulate the state of intestinal microbiota and metabolic state.

**Fig. 8:**
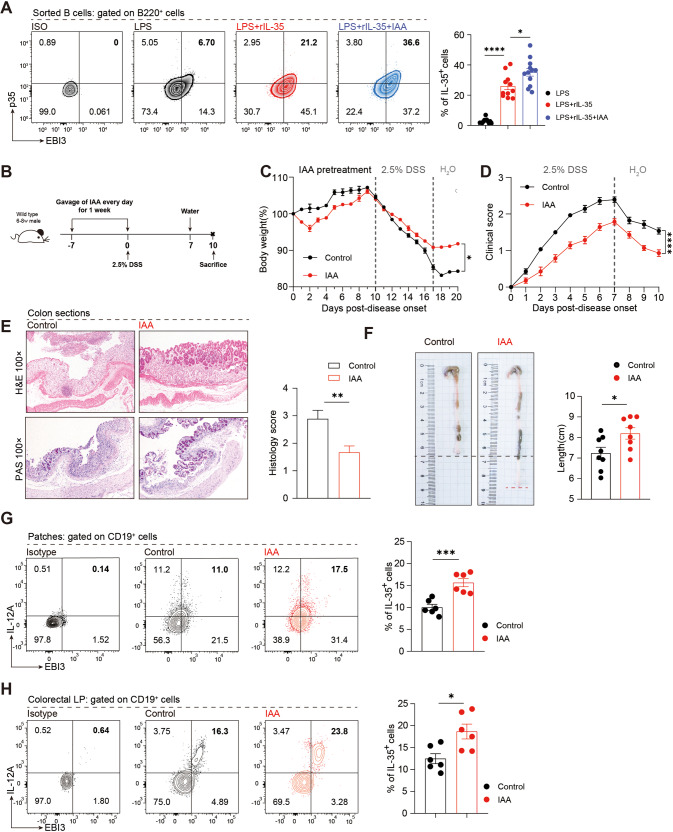
Supplementation with IAA suppresses colitis by skewing the B cell compartment in favor of an IL-35 producing B cell phenotype. **A** Percentage of IL-35 producing B cells (B220^+^ IL-12A^+^ EBI3^+^) in B cells with or without 1000 nM IAA treatment was detected by flow cytometry (lipopolysaccharide (LPS) group *n* = 12; LPS + rIL-35 group *n* = 11; LPS + rIL-35+IAA group *n* = 12). Bar graphs represent the frequencies of IL-35 producing B cells in the groups (right panel). **B** Wildtype (WT) mice were gavaged with IAA daily 1 week prior to the induction of colitis and throughout the experiment. **C** Body weight was calculated (*n* = 4 per group). **D** Disease activity index (DAI) was calculated (*n* = 7 per group). **E** Representative colon sections stained with hematoxylin and eosin (upper panel) and periodic acid-Schiff (lower panel) are shown for each group. Images are shown at ×100 magnification (*n* = 8 per group). **F** Representative colon sections are shown for each group. Length of the colon is shown on the bar graph (*n* = 9 per group). **G**, **H** Cells were isolated from Peyer’s patches (Patches) **G** (*n* = 6 per group) and lamina propria (LP) (**H**) (*n* = 6 per group) of the control or IAA treatment group and the percentage of the IL-35-producing B cells (CD19^+^ IL-12A^+^ EBI3^+^) in B cells was determined by flow cytometry. **C**, **D** Two-way analysis of variance (ANOVA) with Dunnett’s multiple comparisons test. **A**, **E**–**H** Two-tailed unpaired Student’s *t*-test. Data are shown as mean ± standard error of the mean (SEM) and are representative of at least three independent experiments (*P < 0.05, **P < 0.01, ***P < 0.001, ****P < 0.0001).

The intestinal microbes can promote the secretion of cytokines such as IL-10, which plays an important role in alleviating inflammation [18, 25–27]. Therefore, according to the results of metabolic measurements (Fig. 7C, D), we selected the substance with the highest relative expression and the most significant change in colitis to verify its function. To clarify the direct relationship between IAA and IL-35 secretion by B cells we stimulated B cells with IAA and the results showed that IAA could stimulate IL-35 secretion by B cells in a concentration-dependent manner within a certain range under different concentration gradients (Fig. S7A). Further studies showed that 1000 µM IAA could induce B cells to differentiate towards the regulatory B cell phenotype and secrete more IL-10 and IL-35 under IL-35 culture conditions (Fig. 8A, S7B). We further confirmed the results in vivo by establishing an IAA supplement experiment. Before the induction of the colitis model, IAA (0.5 mg/mouse/day) was administered by gavage for seven consecutive days (Fig. 8B), and the changes in colitis progression were closely monitored. Body weight loss (Fig. 8C), DAI score (Fig. 8D), pathological changes of intestinal tissue (Fig. 8E), and gross pathology of the intestines (Fig. 8F) suggested that IAA treatment rescued the disease. Subsequently, flow cytometry was performed to detect the expression of IL-35^+^B cells in the intestinal tissues (Fig. 8G, H). The results showed that the GALT IL-35^+^B cells of IAA treatment group were significantly upregulated, suggesting that IAA can induce the production of IL-35^+^B cells in vivo, and play a role in alleviating colitis. Taken together, IL-35^+^B cells can interact with intestinal microbes to cooperatively maintain intestinal homeostasis.

## Discussion

In this study, through in vivo and in vitro experiments and high-throughput sequencing methods, we demonstrated that IL-35^+^B cells alleviate IBD by interacting with the microbial metabolite IAA in the IBD microenvironment. Moreover, IL-35^+^B cells alleviate the disease by secreting IL-35 which promotes its own expansion through STAT3.

Our previous studies have shown that B cells play a crucial role in the remission of autoimmune diseases such as IBD [28, 29]. When B cells are depleted in vivo, the disease deteriorates irretrievably. Cytokines play a vital role in B-cell disease remission. Although IL-10 has been reported to play an important role in diseases, there is increasing evidence that other cytokines are also essential [30]. Thus, we searched the GEO database and found that EBI3 was elevated. EBI3 and IL-12a form IL-35, while EBI3 and P28 form IL-27. The peripheral blood and colon samples of patients with UC were collected, and an increase of IL-35 but not IL-27 (data not shown) in B cells was observed, indicating that IL-35 may be involved in the process of UC.

We hypothesized that B cells can rescue colitis dependent on IL-35; therefore, we attempted to knock down the IL-35-related molecules. IL-35 is a dimer, consisting of IL-12a and EBI3. Because IL-12a and P40 constitute IL-12, and EBI3 and P28 constitute IL-27 [10, 31], the double-knock mice would have too many deletions of the involved cytokines and the mice did not survive. Therefore, the existing reports all use either EBI3 or IL-12a knockout mice to establish the disease models and demonstrate the role of IL-35 [5, 32]. We found that mice with either EBI3 or IL-12a knockout did not have a more severe disease presentation, suggesting that these molecules themselves cannot solely alleviate the disease. It is worth noting that the disease improved after IL-12a knockdown, which indicates that IL-12 may play a pathogenic role. Interestingly, B cell-derived EBI3 and IL-12a knockdown significantly aggravated the disease, indicating that B cell-derived IL-35 has an alleviatory effect, which is consistent with other reports [4, 5, 33].

We then attempted to adoptively transfer IL-35^+^B cells into mice. IL-35 can induce large amounts of IL-35 in B cells, and about 20% of the intestinal B cells can differentiate into IL-35^+^B cells, but because there is no specific surface marker of IL-35^+^B cells they cannot be isolated. Because rmIL-35 can promote the expansion of many IL-35^+^B cells in vitro and in vivo, we injected exogenous rmIL-35 intraperitoneally to observe the therapeutic effects. We have performed studies regarding the mechanisms of B cells function [28, 34, 35], such as cooperating with Tregs, producing of HSP70 and IgA, etc. Here we aimed to reveal how IL-35^+^B cells work. When intestinal B cells were treated with rmIL-35 in vivo and in vitro it was found that IL-35^+^B cells were expanded, and because they continue to self-promote and expand further, IL-35^+^B cells are sufficient to alleviate the disease during the course of infection. The molecular pathway by which B cell-derived IL-35 function with other cells has not been extensively reported. We used RNA-seq and determined that the expression of the STAT family was significantly increased in cells stimulated with IL-35, especially STAT3 expression, and STAT3 phosphorylation levels were significantly increased which was consistent with other reports [11, 36].

Microbial therapy is the future of medical biology [37], and IAA was reported to promote IL-35^+^B regulatory cells [18]. Therefore, we explored whether the relationship between IL-35^+^B cells and microbes with their metabolites in the intestines could interact to maintain the immune system and intestinal homeostasis. We found that the expression of microbial diversity and the metabolite IAA decreased significantly during the acute phase of colitis and increased during the remission phase. These results confirmed that there were significant differences in the composition of intestinal microbiota in mice during the different stages of colitis, which was often a key factor affecting the outcome of the disease. Importantly, there was a significantly increased expression of IAA observed when exogenous IL-35 was administered, and IAA also promoted IL-35^+^B cell differentiation and expansion. Thus, we elucidated the interaction between IL-35^+^B cells and IAA in maintaining intestinal homeostasis.

## Methods and material

### Human subjects

A total of 14 UC patients and 11 age-matched healthy individuals were recruited at the Department of Gastroenterology of Jing’an District Central Hospital of Shanghai, China with the protocol approved by ethics committee of Jing’an District Central Hospital of Shanghai (Approval number: 2022-018). UC was diagnosed in accordance with The European Crohn’s and Colitis Organization guidelines [38]. Peripheral blood was obtained from 14 UC patients and formalin fixed paraffin-embedded human colon specimens from 6 UC patients were randomly collected at Jing’an District Central Hospital of Shanghai, China. All patients and healthy volunteers enrolled in this study provided informed consents. Detailed Patient information is listed in Table 1.

**Table 1:** Sample Information: Gender, age, patient or Healthy control, stage of disease.

Subject	Gender	Age (years)	Classification	Stage
1	F	21	UC	Active
2	F	24	UC	Active
3	M	56	UC	Active
4	F	62	UC	Active
5	M	58	UC	Active
6	M	43	UC	Active
7	M	33	UC	Active
8	M	25	UC	Active
9	M	65	UC	Active
10	F	34	UC	Active
11	F	57	UC	Active
12	F	19	UC	Active
13	M	65	UC	Active
14	M	48	UC	Active
15	M	42	Healthy control	/
16	M	56	Healthy control	/
17	F	28	Healthy control	/
18	F	24	Healthy control	/
19	F	31	Healthy control	/
20	M	25	Healthy control	/
21	F	21	Healthy control	/
22	F	20	Healthy control	/
23	M	38	Healthy control	/
24	M	32	Healthy control	/
25	F	64	Healthy control	/
26	M	59	Healthy control	/
27	F	40	Healthy control	/
28	M	65	Healthy control	/

### Mice

Wild-type (WT) C57BL/6 mice purchased from SLRC Laboratory Animal (Shanghai, China) were used in this study. B6.129×1-Ebi3tm1Rsb/J(*Ebi3*^−/−^), B6.129S1-Il12atm1Jm/J(*Il12a*^−/−^) and B6.129S2-Ighmtm1cng/J (μMT) mice were purchased from Jackson Laboratory (Bar Harbor, ME, USA). Prior assessments were conducted based on our experience performing survival rate of colitis model under different doses of DSS and other experiments included in this work. All animal procedures in this study were approved by the animal care and use committee of Fudan University and conformed to the Guide for the Care and Use of Laboratory Animals.

### Dextran sodium sulfate (DSS)-induced Colitis

Experimental colitis was established by administering 2.5% DSS (w/v; MP Biomedicals, Irvine, CA, USA) in the drinking water for 7 days as previously described [39], normal drinking water was then given for 3 days in order to mimic the remission phase of ulcerative colitis.

### Adoptive transfer

B cells were purified from the spleens of WT mouse or IL-35 gene knockout (*Il12a*^−/−^ and *Ebi3*^*−/−*^) mouse by using a mouse B cell negative isolation kit (StemCell Technologies, Vancouver, BC, Canada). Purified B cells were adoptively transferred into 8-week-old male μMT mice intravenously, followed by the administration of 2.5% DSS. Adoptive transfer was performed with 1 × 10^7^ cells/mouse 48 h before DSS administration.

### IL-35 in vitro rescue assay

Recombined mouse IL-35(rIL-35) was purchased from Chimerigen (Liestal, Switzerland). WT mice were randomly assigned to four groups: (1) Isotype; (2) rIL-35; (3) Isotype+DSS induction and (4) rIL-35+DSS induction. In group (2) and group (4), rIL-35 was administered intravenously [0.75 μg/mice, dissolved in 200 μl phosphate-buffered saline (PBS)] to the mice 48 h before model induction. Isotype groups [group (1) and group (3)] were received IgG administration [0.75 μg/mice, dissolved in 200 μl phosphate-buffered saline (PBS)].

### Microbiota transplantation

To eliminate gut microbiota, Wild-type mice were treated with antibiotic cocktail ABX (1 g/L metronidazole; 0.5 g/L vancomycin; 1 g/L ampicillin; 1 g/L neomycin) as described previously for 14 days (ABX mice). Feces of mice treated with rIL-35 or isotype were collected on day 7 and then resuspended in 5% thioglycollate broth (w/v, Solarbio, Beijing, China). Fecal mixture was centrifuged at 2000 rpm, supernatant was collected. The supernatant was orally gavaged into ABX mice for 7 days fecal transplantation, mice were then administered with 2.5% DSS to induce colitis.

### Gavage with IAA

Wild-type mice were orally gavaged with IAA (0.5 mg/mice, dissolved in oil) daily 1 week prior to the induction of colitis and throughout the experiment. Control group mice received oil only.

### Histopathology

Colons were flushed using PBS and then fixed in 4% paraformaldehyde and embedded in paraffin. Colon sections were stained with hematoxylin & eosin (H&E) or Periodic Acid Schiff (PAS). Histological scoring of colitis was performed by at least two pathologists under a blinded fashion. The score was a combination of tissue Integrity and inflammatory cell infiltration.

### Immunofluorescence staining

Peyer’s patches and human colon biopsy samples were collected and fixed in paraformaldehyde and embedded in paraffin. For immunofluorescence (IF) staining, tissue sections were blocked with 5% (w/v) fetal bovine serum (FBS) for 30 min at room temperature, and then stained with purified antibodies for CD20 (for mice samples: 1:100, Abcam, Cambridge, MA, USA, ab271288; for human samples: 1:100, Abcam, ab279300), IL-12A (1:100, Abcam,ab131039) and EBi3 (1:50, Santa Cruz Biotechnology, Santa Cruz, CA, USA) for 15 h at 4 °C, and then stained with Alexa Fluor 488 anti-rabbit IgG monoclonal antibody (Thermo Fisher Scientific, Waltham, MA, USA), Alexa Fluor 568 anti-rat IgG monoclonal antibody (Thermo Fisher) and Alexa Fluor 647 anti-mouse IgG monoclonal antibody (Thermo Fisher) for 1 h at room temperature. The specmens were analysed using fluorescence microscopy.

### Cell isolation and differentiation

B cells were purified from the spleens of WT mouse by using a mouse B cell negative isolation kit (StemCell Technologies). sorted naive B cells were then stimulated for 72 h with 10 µg/mL LPS (Sigma-Aldrich, St. Louis, MO, USA).

For IL-35 producing B cell differentiation, naive B were cultured under IL-35 producing B cell culture system with 10 µg/mL LPS and 20 ng/mL rmIL-35 for 72 h.

### Real-time polymerase chain reaction (PCR) analysis

Total RNA of B cells was extracted using RNA extraction kit (Tiangen Scientific, Beijing, China) followed reverse transcription. Real-time polymerase chain reaction was performed using SYBR Green Master Mix (TaKaRa, Tokyo, Japan) on the ABI 7500 Thermocycler (Applied Biosystems, California, USA) according to the manufacture’s instructions. The relative gene expression compared with GAPDH was calculated using the ΔΔCt method. Primers for target genes are listed in Table 2.

**Table 2:** Primer Sequence: Primer sequence of mice genotyping and qRT-PCR.

Primer	Sequence(5′-3′)	Usage
muMT Mutant R	TTGTGCCCAGTCATAGCCGAAT	Mice genotyping
muMT common	CCGTCTAGCTTGAGCTATTAGG	Mice genotyping
muMT WT R	GAAGAGGACGATGAAGGTGG	Mice genotyping
IL12A Mutant F	CTGAATGAACTGCAGGACGA	Mice genotyping
IL12A Mutant R	ATA CTT TCT CGG CAG GAG CA	Mice genotyping
IL12A Wild Type F	CAG CAT GTG TCA ATC ACG	Mice genotyping
IL12A Wild Type R	TCA CCA TGT CAT CTG TGG	Mice genotyping
EBi3 Mutant R	GCC AGA GGC CAC TTG TGT AG	Mice genotyping
EBi3 Common	AAC CTC AGG CCA GGC AGT	Mice genotyping
EBi3 Wild Type R	TTC CGT AGG CCA TGT AGG AC	Mice genotyping
IL12p35 F	GACCTGTTTACCACTGGAACTA	qRT-PCR
IL12p35 R	GATCTGCTGATGGTTGTGATTC	qRT-PCR
EBi3 F	CTTCTCTCTCAAGTACCGACTC	qRT-PCR
EBi3 R	TTATGGGGTGCACTTTCTACTT	qRT-PCR
β-actin F	CCAGCCTTCCTTCTTGGGTATG	qRT-PCR
β-actin R	TGTGTTGGCATAGAGGTCTTTACG	qRT-PCR

### Western blot

B cells were negatively purified from WT mice and cultured under IL-35 producing B cells’ culture system. Then B cells were lysed and protein from 1 × 10^7^ cells was resolved by SDS-PAGE and transferred to polyvinylidene fluoride (PVDF) membranes (Merck Millipore, Billerica, MA, USA), and blotted with anti-STAT3 (1:5000, Abcam) and anti-p-STAT3 (1:5000, Abcam). Bound antibodies were revealed with HRP-conjugated species-specific secondary antibodies using ECL substrate (Amersham Pharmacia Biotech, Station, NY, USA).

### Enzyme-linked immunosorbent assay

Fecal supernatant was collected from colitis mice on days 0, 4, 7, and 10. IL-6, TNF-α, interferon (IFN)-γ, IL-10, IL-35 and immunoglobulin A (IgA) levels in the supernatants were determined by enzyme-linked immunosorbent assay (ELISA) according to the manufacture’s protocols (Thermo Fisher Scientific).

### Measurement of IAA in Mouse Feces

Fecal supernatant was collected from colitis mice treated with rIL-35 or isotype at day 10. The contents of IAA in the fecal samples were determined with ELISA kit from Shanghai ShuHua Biological Technology Co. Ltd. (Shanghai, China), according to the manufacture’s instruction.

### Flow cytometry

For surface marker staining, mononuclear cells were previously blocked with anti-CD16/32 antibody (Thermo Fisher Scientific) for 15 min and then incubated with the following anti-mouse antibodies: PE-CD19, PE-B220 and Pacific blue-CD45 (all purchased from Thermo Fisher Scientific).

For intracellular marker staining, cells were previously stimulated with 50 ng/mL PMA and 750 ng/mL ionomycin in the presence of 20 µg/mL brefeldin A for 5 h, then surface-stained for 30 min. After surface marker staining, cells were resuspended in Fixation/Permeabilization Buffer Set (Thermo Fisher Scientific) and stained with PE-Cy7-IL-10 (BioLegend, San Diego, CA, USA); APC-IL-12A (Thermo Fisher Scientific) and FITC-EBI3 (Novus Biologicals, Littleton, CO, USA) for 45 min according to the manufacturer’s protocol.

For intranuclear (p-STAT3) marker staining, cells were previously surface stained and then fixed and permeabilized using a transcription factor staining buffer set (Thermo Fisher Scientific) and stained with PE-p-STAT3 (Thermo Fisher Scientific) for 45 min according to the manufacturer’s protocol.

Isotype antibodies were used as negative controls. Flow cytometry analysis was performed using BD Celesta Analyzer.

### RNA sequencing

Patches’ CD19^+^ B cells were isolated from wild type mice at day 0, 4, 7 and 10. Total RNA was isolated from sorted B cells using the Total RNA isolation kit (Tiangen Scientific) according to manufacturer’s instructions. And then total RNA was qualified and quantified with a Nano Drop and Agilent 2100 bioanalyzer (Thermo Fisher Scientific) followed by mRNA purification.

A total of 500 ng purified mRNA was fragmented followed by reverse transcription and second strand cDNA synthesis. The double strand PCR products above were heated denatured and circularized by the splint oligo sequence. The final library was amplified with phi29 (Thermo Fisher Scientific) and single end 50 bases reads were generated on BGISEQ500 platform (BGI, Shenzhen, China). Reads were then mapped to the Mus musculus GRCm38 reference genome. Gene annotations were applied from Ensembl. Gene expression levels were quantified using htseq-count and estimated independently with TPM with Kallisto.

### 16 S rDNA sequencing

24 samples of fecal material from Wild-type mice at day 0, 4, 7 and 10 after 2.5% DSS administration was extracted using the CTAB kit according to manufacturer’s protocol. Total DNA was eluted in Elution buffer and barcoded primers[341 F(5’-CCTACGGGNGGCWGCAG-3’);805 R(5’-GACTACHVGGGTATCTAATCC-3’)] were designed to span the V3-V4 region of 16 S rRNA gene as previously described. The PCR conditions was consisted of an initial denaturation at 98 °C for 30 s; 32 cycles of denaturation at 98 °C for 10 s, annealing at 54 °C for 30 s, and extension at 72 °C for 45 s; and then final extension at 72 °C for 10 min. The amplicon pools were prepared for sequencing and the size and quantity of the amplicon library were assessed on Agilent 2100 Bioanalyzer and with the Library Quantification Kit for Illumina, respectively. The libraries were sequenced on NovaSeq PE250 platform. Samples were sequenced on an Illumina NovaSeq platform according to the manufacturer’s recommendations.

### Analysis of tryptophan metabolites by high performance liquid chromatography

For High Performance Liquid Chromatography analysis of tryptophan metabolites, feacal samples were collected and froze in liquid nitrogen before metabolites measurement. The metabolites were extracted from feacal samples using high throughput tissue lyser according to the manufacture’s protocol. Then extracted metabolites were resolved using ACQUITY UPLC® HSS T3 Column (2.1×150 mm, 1.8 μm, Waters, Watertown, MA, USA) on a ACQUITY analysis system from Waters and identified using Lipid Search software.

### Data accession

The raw data of RNA sequencing are available in GEO (http://www.ncbi.nlm.nih.gov/geo/) with the accession number GSE210506. And the accession number for the 16 S rRNA sequencing reported in this paper is PRJNA866337.

### Statistical analysis

Quantitative data were expressed as mean ± Standard Error of Mean (SEM). Differences were determined by unpaired two-tailed *t*-test for comparing two groups. For murine clinical data (body weight and clinical scores), differences among different groups were determined using two-way ANOVA (Dunnett’s multiple comparisons test). Correlations of 32 representative gut microbiota species and 21 representative tryptophan metabolites were evaluated using Spearman’s rank correlation coefficient. All *p* < 0.05 were considered significant. Statistical tests were carried out using GraphPad Prism (La Jolla, CA, USA, USA) v. 8.0.1 Software.

## Supplementary information

supplementary figures

full length uncropped original WB

full length uncropped original WB

full length uncropped original WB

## Data Availability

The authors confirm that the data supporting the findings of this study are available within the article and its supplementary materials.
