# Supplementary material for: Interleukin-35 -producing B cells rescues inflammatory bowel disease in a mouse model via STAT3 phosphorylation and intestinal microbiota modification
Source: Cell Death Discov. 2023 Feb 17;9:67. doi: 10.1038/s41420-023-01366-5 (PMC9935866; doi:10.1038/s41420-023-01366-5)
Supplement: Supplementary file 1 — supplementary figures [file 41420_2023_1366_MOESM1_ESM.pdf]

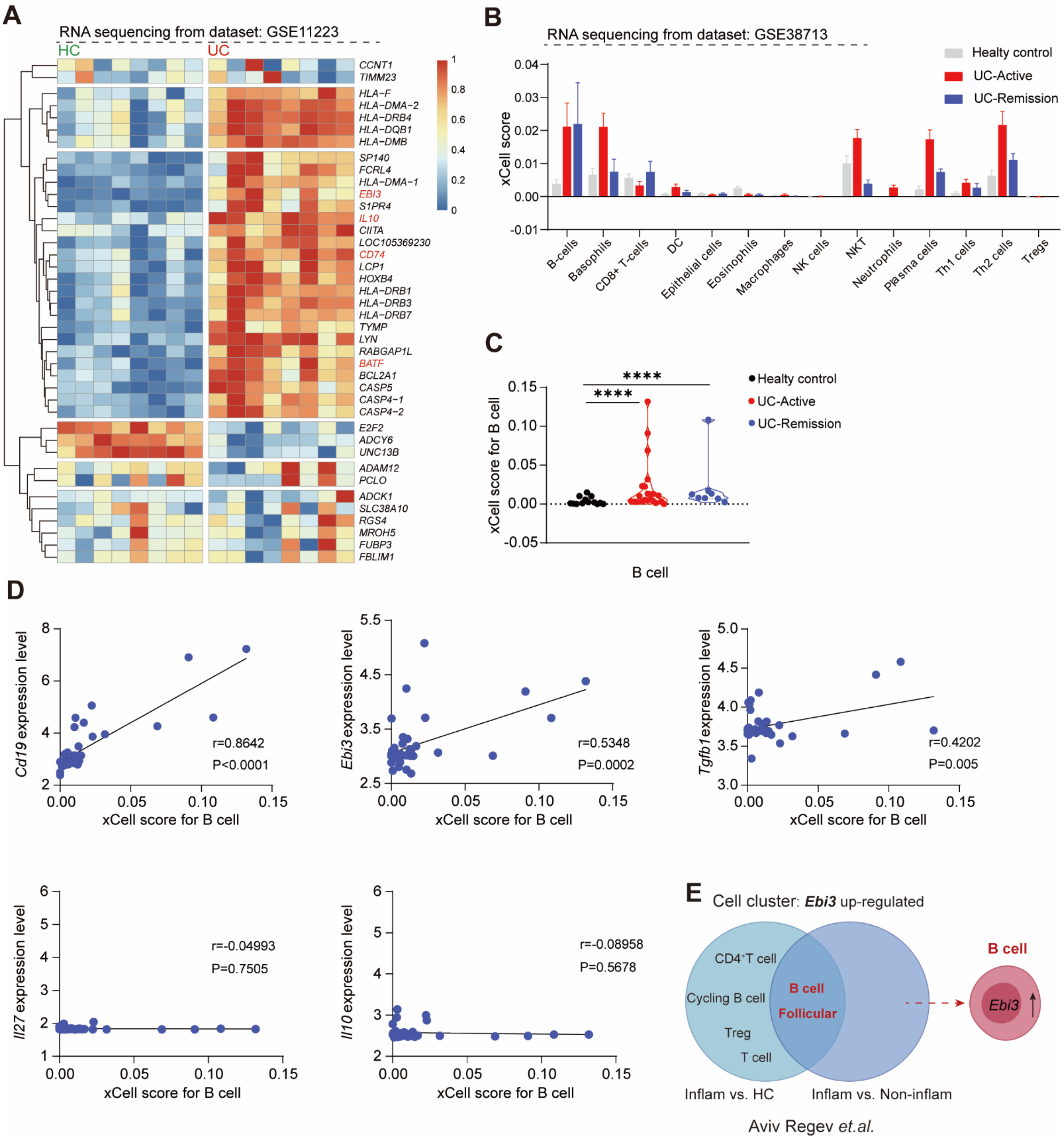

Figure S1 IL-35 producing B cells related genes up-regulated in human colitis samples

(A) RNA sequencing data from GEO datasets: GSE11223 (human colon biopsy samples collection) was downloaded and analyzed. 8 Healthy control and 8 UC patient samples were randomly selected from datasets. Heatmap shows the expression level of top differential expression genes between UC group and HC group. Expression level of genes were normalized and shown in the form of TPM.

(B) The gene expression profiling by array (GSE38713) was obtained from the GEO database. Immune cell proportions in the colon of healthy controls and ulcerative colitis patients were analyzed using xCell (Healthy

control group n=13; UC patient in active stage n=22; UC patient in remission stage n=8).

(C) B cell proportions in the colon of healthy controls and ulcerative colitis patients were analyzed using xCell .

(D) Correlation between Breg related genes and xCell score of B cells were analyzed.

(E) Venn diagram shows the immune cell populations which EBi3 up-regulated significantly during the acute stage of ulcerative colitis. The differential expression genes of scRNA sequencing data were download from a published literature (PMID: 31348891).

(B) Unpaired Student's t test with two-tailed.

(C-D) Pearson correlation coefficient.

Data are shown as mean±SEM and are representative of at least 3 independent experiments (*P < 0.05, **P < 0.01, ***P<0.001, ****P<0.0001).

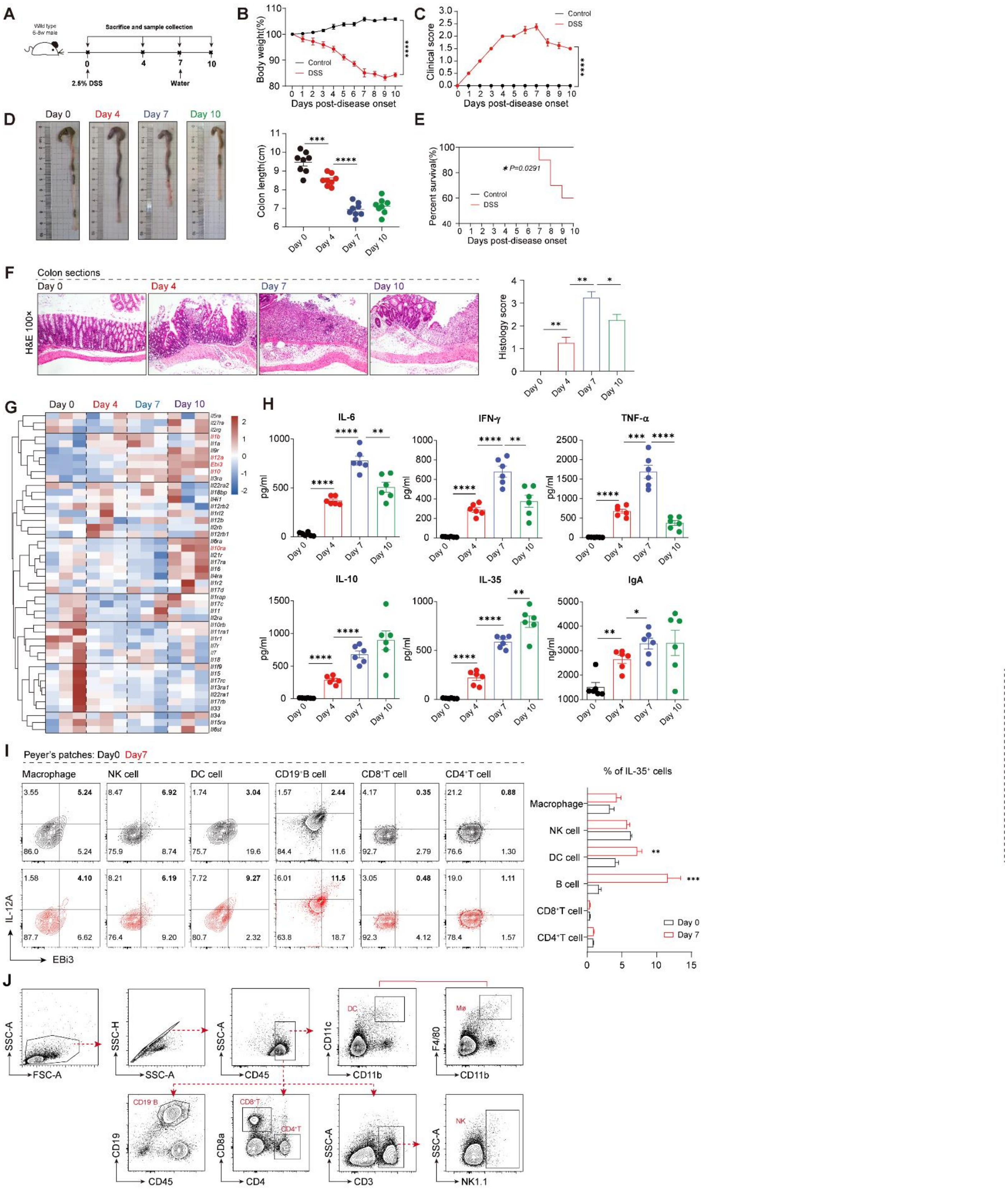

Figure S2 IL-35 producing B cells play an important immune regulatory role in colitis and B cells are main resource of IL-35

(A) Wild-type mice were administrated with 2.5%DSS and sacrificed on day 0,4 ,7 and 10 post-disease onsets.

(B) Body weight was calculated (n=4 in each group).

(C) Clinical score was calculated (n=4 in each group).

(D) Representative colon sections are shown for each group (left panel). Length of the colon is shown on the

bar graph (right panel) (n=8 in each group).

(E) Survival analysis of each group was calculated (n=10 in each group).

(F) Representative colon sections stained with H&E are shown for each group. Images are shown at×100 magnification (n=4 in each group).

(G) Heatmap shows the expression of cytokine and receptor related genes in $CD19^+$ B cells isolated from Peyer's patches of colitis mice at different post-disease onsets. Data were normalized and shown in the form of TPM (n=6 in each group).

(H) Fecal supernatant was collected from colitis mice at day 0, 4, 7 and 10 post-disease onsets. The production level of pro-inflammatory cytokines: IL-6, IFN-γ, TNF-α, and anti-inflammatory cytokines: IL-10, IL-35 and IgA were detected with ELISA (n=6 in each group).

(I) Cells were isolated from Peyer's patches of colitis mice at day 0 and day 7 post-disease onsets and the immune landscape of IL-35 producing cells in these cells were analyzed using flow cytometry (n=6 in each group). To distinguish different cell populations, the FACS panel was designed as below: $CD45^+CD11b^+F4/80^+$ Macrophage cells, $CD3^-NK1.1^+$NK cells, $CD45^+CD11b^+CD11c^+$ DC cells, $CD3^+CD8^+$ T cells, $CD3^+CD4^+$ T cell, $CD19^+$ B cells. The bar plot (right) shows the percentage of IL-35 in different immune cells.

(J) Gate strategy of immune cell subsets.

(B, C) Two-way ANOVA with Dunnett's multiple comparisons test.

(D, F, H, I) Unpaired Student's t test with two-tailed.

(E)Log-rank (Mantel-Cox) test.

Data are shown as mean±SEM and are representative of at least 3 independent experiments (*P < 0.05, **P < 0.01, ***P<0.001, ****P<0.0001).

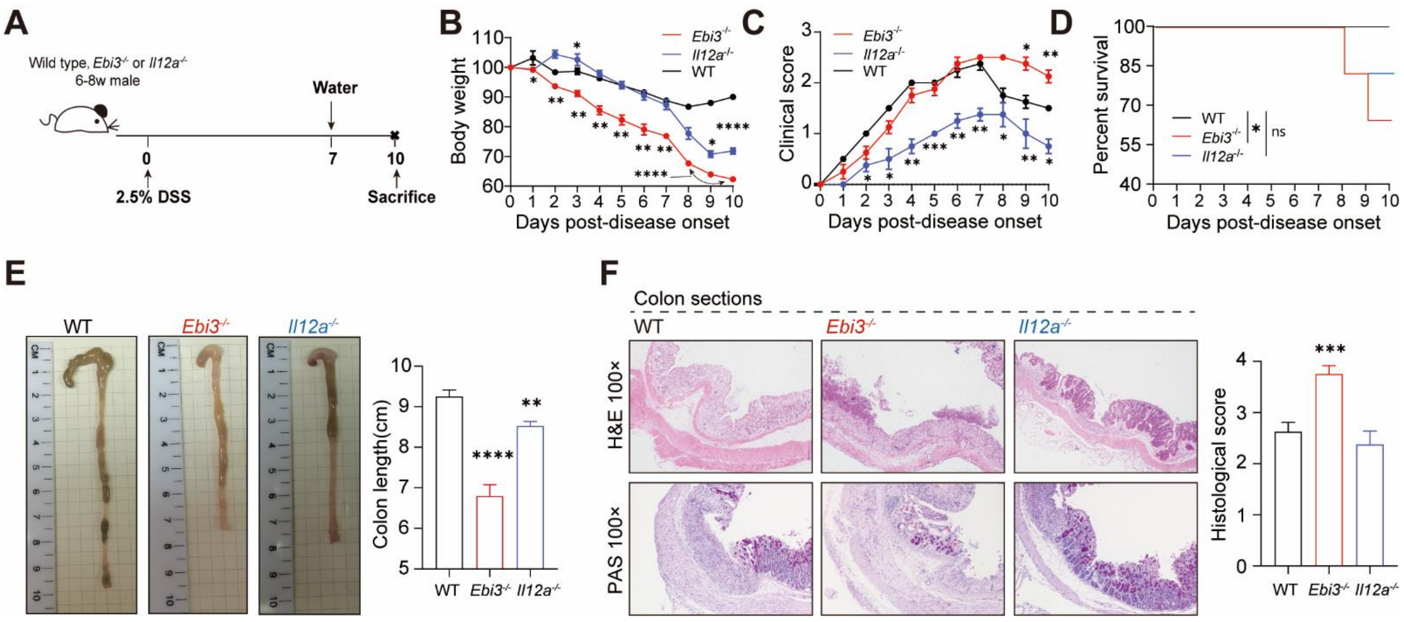

Figure S3 IL12A or EBi3 nonspecific knockout leads to different phenotype in DSS-induced colitis

(A) *$Il12a^{-/-}$*, *$Ebi3^{-/-}$* and WT mice were administrated with 2.5% DSS.

(B) Body weight was calculated (n=4 in each group).

(C) Clinical score was calculated (n=4 in each group).

(D) Survival analysis of each group was calculated (n=8 in each group).

(E) Representative colon sections are shown for each group (left). Length of the colon is shown on the bar graph (right) (n=8 in each group).

(F) Representative colon sections stained with H&E (above) and PAS (below) are shown for each group. Images are shown at×100 magnification. Histological sections were blindly scored on a scale of 0 to 4 to generate a histological score and individual mouse scores are shown with each data point representing a single mouse (n=8 in each group).

(B, C) Two-way ANOVA with Dunnett's multiple comparisons test.

(E, F) Unpaired Student's t test with two-tailed.

(D)Log-rank (Mantel-Cox) test.

Data are shown as mean±SEM and are representative of at least 3 independent experiments (*P < 0.05, **P < 0.01, ***P<0.001, ****P<0.0001).

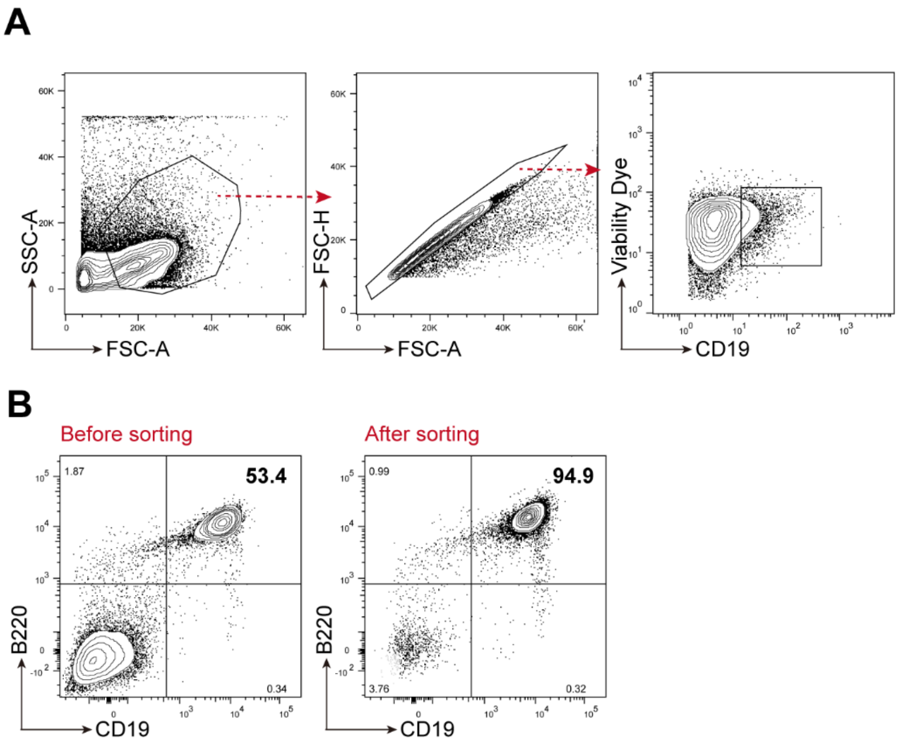

Figure S4 B cell sorting

(A) Gate strategy of B cells.

(B) B cell expression before and after sorting.

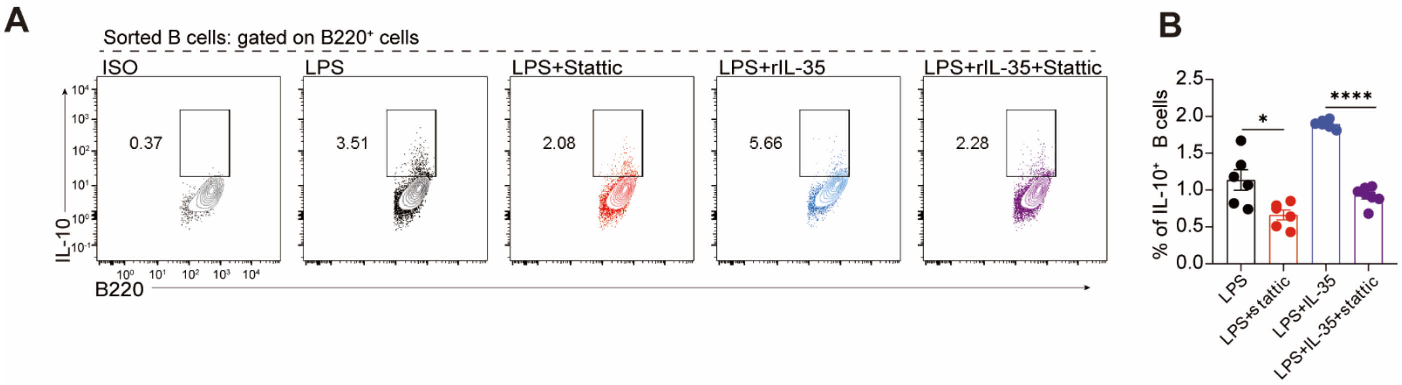

Figure S5 Inhibition of STAT3 phosphorylation leads to reduction of IL-10 producing B cell population

(A) Percentage of IL-10 producing B cells (B220$^+$ IL-10$^+$ cells) under IL-35 producing B cells culture system with or without 10nM STAT3 inhibitor (stattic) was detected by flow cytometry (n=6 in each group).

(B) Dots represent B220$^+$ IL-10$^+$ cell frequencies in groups. Unpaired Student's t test with two-tailed.

Data are shown as mean±SEM and are representative of at least 3 independent experiments (*P < 0.05, **P < 0.01, ***P<0.001, ****P<0.0001).

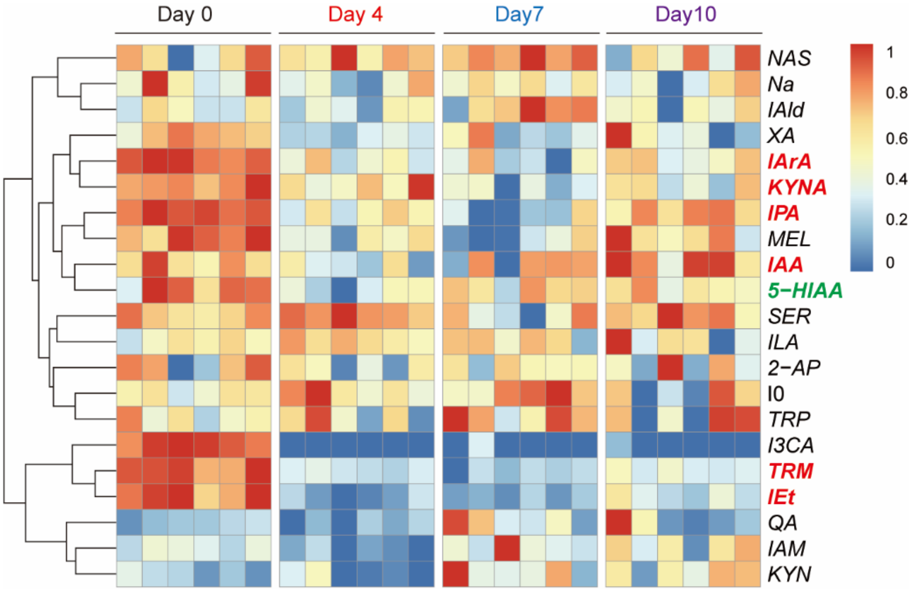

Figure S6 Expression of Tryptophan metabolites in gut at different time point post colitis induction

Heatmap shows expression level of tryptophan metabolites in 24 fecal samples from colitis mice at different post-disease onsets (n=6 in each group).

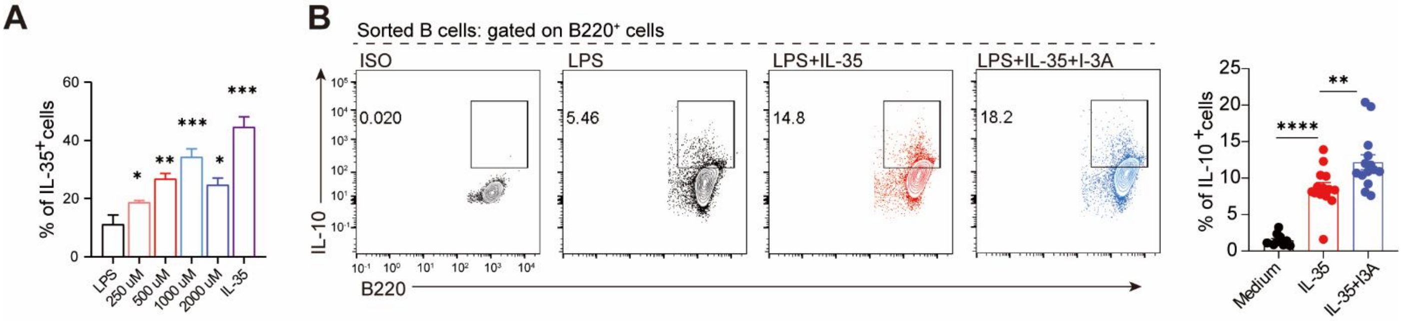

Figure S7 IAA treatment induces IL-10 producing B cell in vivo

(A) Percentage of IL-35 producing B cells ($B220^{+}$ $IL\text{-}12A^{+}$ $EBI3^{+}$) in B cells under different stimulation (n=4 in each group). Two-way ANOVA with Dunnett's multiple comparisons test.

(B) Percentage of IL-35 producing B cells ($B220^{+}$ $IL\text{-}12A^{+}$ $EBI3^{+}$) in B cells with or without 1000 nM IAA treatment was detected by flow cytometry (n=14 in each group). Unpaired Student’s t test with two-tailed.

Data are shown as mean±SEM and are representative of at least 3 independent experiments (*P < 0.05, **P < 0.01, ***P<0.001, ****P<0.0001).
