# Supplementary figures and images for: Interleukin-35 -producing B cells rescues inflammatory bowel disease in a mouse model via STAT3 phosphorylation and intestinal microbiota modification
Source: Cell Death Discov. 2023 Feb 17;9:67. doi: 10.1038/s41420-023-01366-5 (PMC9935866; doi:10.1038/s41420-023-01366-5)

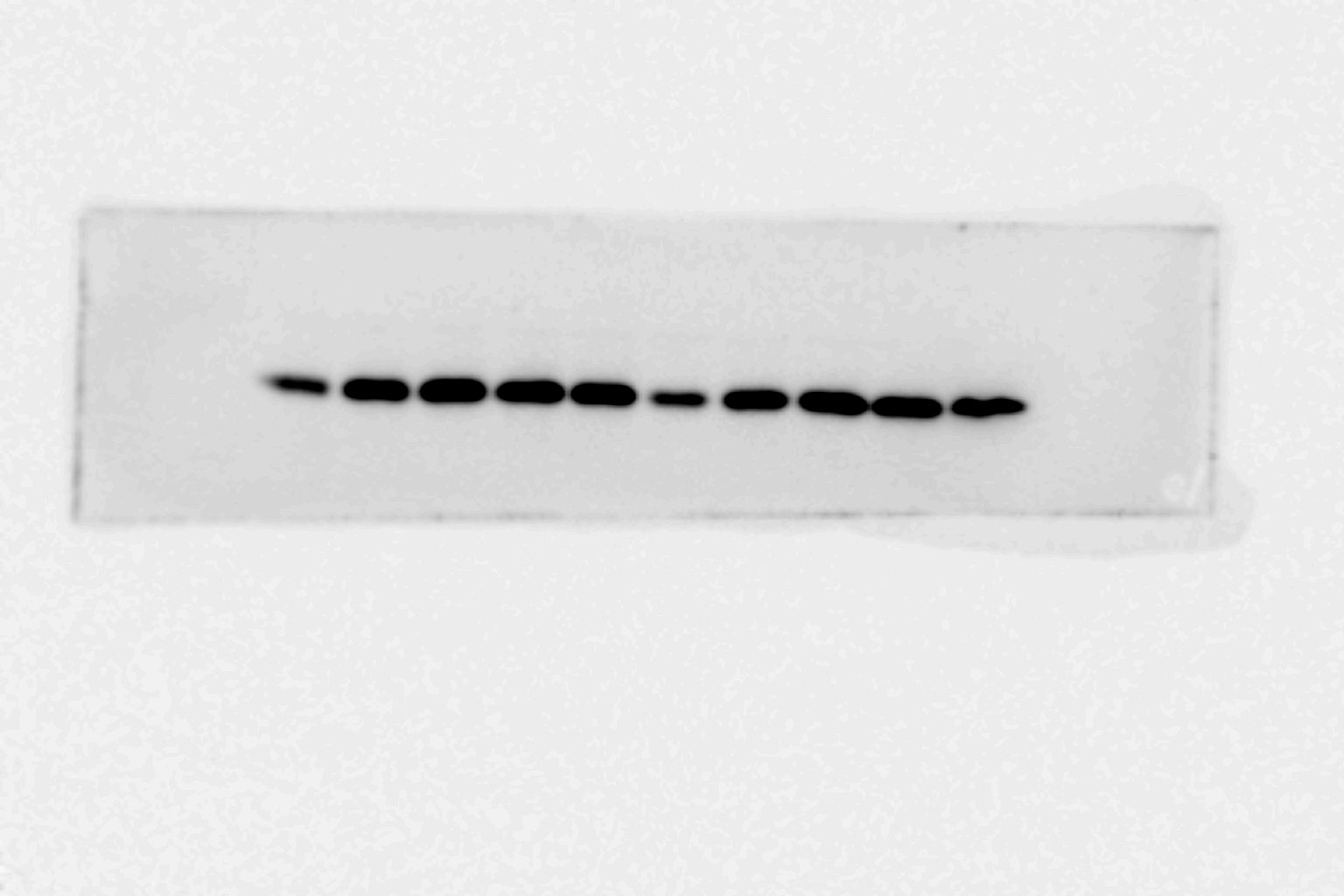

Supplement: Supplementary file 2 — full length uncropped original WB [file 41420_2023_1366_MOESM2_ESM.tif]

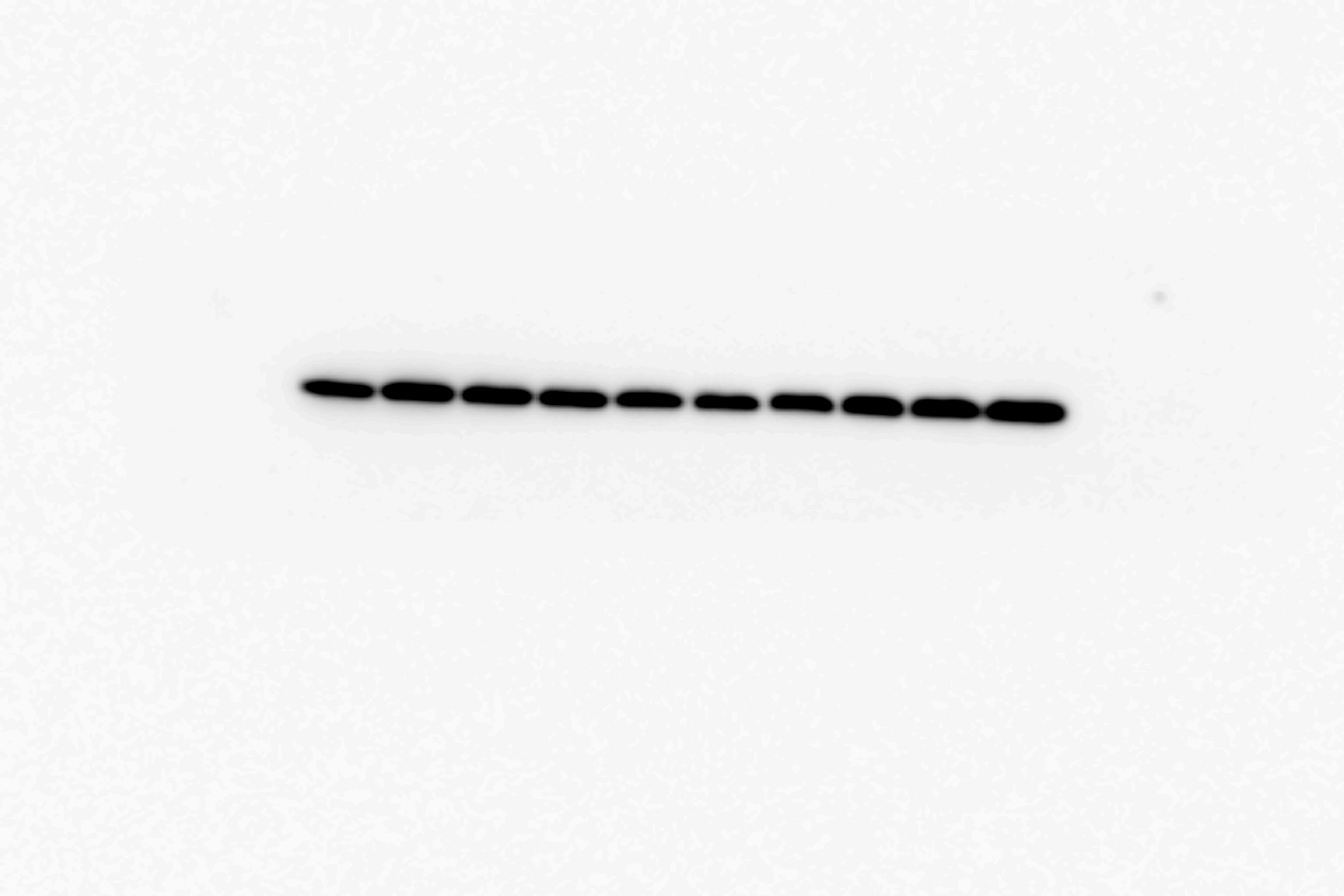

Supplement: Supplementary file 3 — full length uncropped original WB [file 41420_2023_1366_MOESM3_ESM.tif]

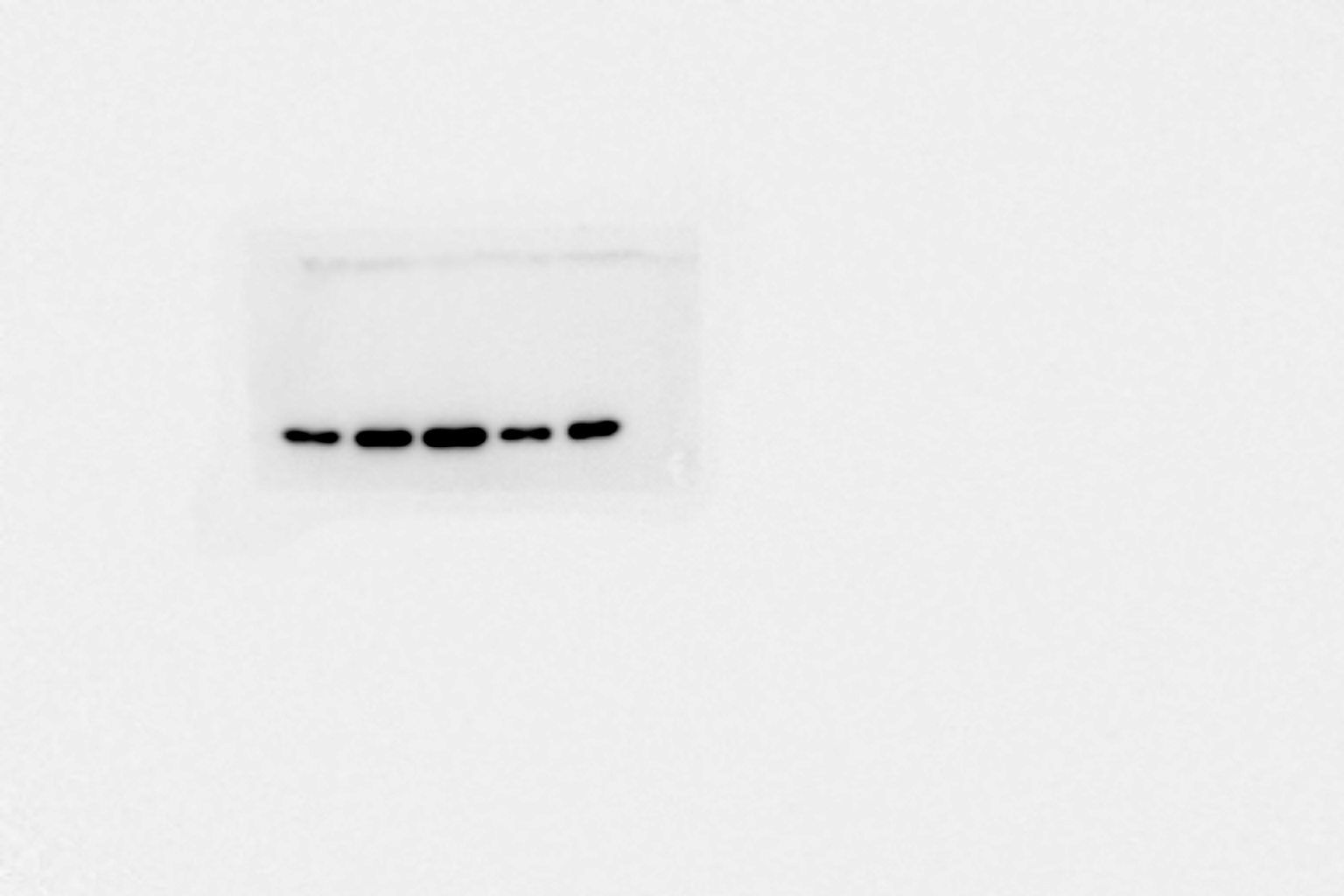

Supplement: Supplementary file 4 — full length uncropped original WB [file 41420_2023_1366_MOESM4_ESM.tif]
